# Perfluorooctanoic Acid (PFOA) Induces Redox Status Disruption in Swine Granulosa Cells

**DOI:** 10.3390/vetsci9060254

**Published:** 2022-05-26

**Authors:** Giuseppina Basini, Simona Bussolati, Veronica Torcianti, Francesca Grasselli

**Affiliations:** Dipartimento di Scienze Medico-Veterinarie, Università di Parma, Via del Taglio 10, 43126 Parma, Italy; simona.bussolati@unipr.it (S.B.); veronica.torcianti@studenti.unipr.it (V.T.); francesca.grasselli@unipr.it (F.G.)

**Keywords:** PFAS, ovary, cell viability, free radicals, oxidative stress

## Abstract

Perfluorooctanoic acid (PFOA) is employed in the production and processing of several plastic materials, mainly during the production of waterproof fabrics or nonstick cookware. PFOA is identified as a substance of very high concern, as it is classified as a persistent, bioaccumulative, and toxic (PBT) substance because of its persistence in the environment and its potential accumulation in organisms. Thus, safe levels of exposure cannot be established, and PFOA emissions should be minimized. PFOA has recently been linked to several health concerns in humans. In particular, a disruptive effect on redox status homeostasis has been documented, with a potential impairment of normal reproductive function that requires adequate oxidative balance. Therefore, the aim of the present study was to evaluate the effects of PFOA (2, 20, and 200 ng/mL) on ovarian granulosa cells, a model of reproductive cells. The obtained results reveal that PFOA stimulated cell viability (*p* < 0.05). Regarding the effects on free radical production, O_2_^−^, NO, and H_2_O_2_ were significantly inhibited (*p* < 0.05), while the nonenzymatic antioxidant power was not significantly modified. Collectively, the present results deserve attention since free radical molecules play a crucial role in ovarian follicle development leading to a successful ovulation.

## 1. Introduction

In recent years, perfluoroalkyl substances (PFAS) have gained increasing attention as an emerging category of pollutants. This family of chemical compounds were produced in the late 1940s through electrochemical fluorination or telomerization. Since their chemical structure renders them stable from a chemical and thermal point of view, and difficult to biodegrade in the environment, they have wide industrial applications; they are used as surfactants and emulsifiers, in the production of other fluorinated chemicals, stain removers, friction reducers, firefighting foams, and waterproofing and insulating agents [1]. Potential routes of human exposure to PFAS include the ingestion of dust present in the environment, inhalation, skin absorption, and the consumption of contaminated drinking water or food [2]. Direct exposure occurs when PFAS are present in food or in house dust, while indirect exposure happens when PFAS are formed through the transformation of fluorotelomeric-based materials, including polyfluoroalkyl phosphate esters (PAP), fluorotelomer acrylate (FTAc), fluorotelomeric iodides (FTI), and fluorotelomeric alcohols (FTOH) [3]. PFAS are particularly resistant to hydrolysis, photolysis, or microbial degradation due to their chemical structure, and they can easily accumulate in organisms. Thus, these compounds are ubiquitous, and their presence was documented in biota and human serum [4]. Perfluorooctanoic acid (PFOA), a well-studied and ubiquitous PFAS, is an eight-carbon fluorocarbon and carboxylic acid group detectable in wild animal serum [5]. Once absorbed by humans, PFOA binds to blood proteins, mainly albumin, and it is not easily metabolized, leading to a half-life of 2.3–8.5 years [6]. Among the various uses of PFAS, PFOA is widely employed for the production of fluoropolymers such as polytetrafluoroethylene (better known as Teflon), of which the nonstick properties and chemical inertness are widely known, and polyvinyldenefluoride (PVDF). A further application of PFOA is represented by Gore-Tex^®^, a resistant waterproof material characterized by breathability and biocompatibility that is used in the production of technical sports clothing and medical-health items, such as vascular prostheses, heart valves, and sutures, and in cosmetic surgery. Experiments performed on animals showed that PFOA mainly accumulates in the liver, kidneys, and serum [7], causing negative health effects, including hepatotoxicity, genotoxicity, immunotoxicity, and neurotoxicity, which raise increasing concerns due to its long half-life. In 2016, it was also classified by the International Agency for Research on Cancer (IARC) as “possibly carcinogenic to humans”, belonging to class 2B. Furthermore, several studies on animal exposure to PFOA have suggested possible interference with endocrine signaling [8]. In particular, a study conducted by Di Nisio et al. showed that PFOA disrupts the male hypothalamus–pituitary–gonadal (HPG) axis [9]. The present work investigates the possible consequences of PFOA on female gonads by means of our well-validated model of endocrine reproductive cells [10,11,12]. In particular, we hypothesized a disruption in the fine balance between free radicals and antioxidant mechanisms induced by PFOA. To this goal, granulosa cells were collected from follicles. The pig was chosen as a valuable animal model for translational medicine, as it is similar to humans from anatomical, genetic, and physiological points of view [13]. The effect of PFOA was studied on granulosa cell viability and on oxidative-stress-related parameters, since redox status is crucial for correct follicular function leading to ovulation [14].

## 2. Materials and Methods

All reagents were purchased from Sigma Chemical Co., Ltd. (St. Louis, MO, USA), while the plastics were from Sarstedt AG & Co. (Numbrecht, Germany). If purchased elsewhere, it is highlighted in the text.

### 2.1. Isolation and Culture of Granulosa Cells

To obtain the granulosa cells, swine ovaries were collected at a local slaughterhouse and placed in a refrigerated container with phosphate-buffered saline (PBS; 4 °C) with the addition of penicillin (100 Ul/mL), streptomycin (100 Ul/mL), and amphotericin B (2.5 µg/mL), stored in a freezer bag, and transported to the laboratory within 1 h. In order to improve cleaning in the laboratory, the ovaries were immersed for 1 min in 70% ethanol and subjected to further washing with PBS [15]; during the selection, ovaries with cystic or hemorrhagic follicles were discarded. Granulosa cells were aspirated from preovulatory follicle with a diameter greater than 5 mm with a 26-gauge needle [16,17]. Cells were then subjected to centrifugation at 450× *g* for 10 min, and the cell pellet was treated with ammonium chloride 0.17M at 37 °C for 1 min to eliminate any red blood cells in the precipitate. Treatment with ammonium chloride could be repeated if a high number of erythrocytes are present. The cell number was counted by viable trypan blue dye (0.4% *w*/*v*). Cells were then plated and cultured in a validated serum-free system composed by DMEM/Ham’s F12 medium supplemented with penicillin (100 µg/mL), amphotericin B (2.5 µg/mL), streptomycin (100 µg/mL), sodium selenite (5 ng/mL) and transferrin (5 µg/mL) [10,18,19], indicated hereafter as CM. This serum-free medium is a culture system designed to keep granulosa cells differentiated and prevent luteinization. At the time of seeding in 96-well plates, cells were treated with PFOA (2, 20, and 200 ng/mL) on the basis of tested concentrations in previous works [20,21]. Cells were then incubated at 37 °C under humidified conditions (5% CO_2_) for 48 h.

#### 2.1.1. Granulosa Cell Viability

The vitality of granulosa cells was evaluated using a bioluminescent assay (ATP-lite; Packard Bioscience, Groningen, The Netherlands). ATP is a marker of cell viability because it is present in all metabolically active cells, while its concentration declines very rapidly in the presence of necrosis or apoptosis; the test is based on light emission due to the reaction among ATP, luciferase, and luciferin. The emitted light is proportional to the ATP concentration. The test was validated by plating different viable cell numbers (from 2.5 × 10^3^ to 4 × 10^6^/100 µL) The curve was repeated three times. The relationship between cell number and luminescence was linear (r = 0.95). Cells were seeded in 96-well plates at a density of 2 × 10^5^ cells/100 µL CM, and treated with PFOA as detailed above. After 48 h of incubation with the treatments, by adding the kit reagents, it was possible to measure luminescence, proportional to the number of viable cells, using the luminometer Victor Nivo (Perkin Elmer, Groningen, The Netherlands) [12]. 

#### 2.1.2. Granulosa Cell Redox Status

##### Granulosa Cell Superoxide (O_2_^−^) Production

O_2_^−^ production was evaluated by WST-1 (4-[3-(4-iodophenyl)-2-(4-nitrophenyl)-2H-5-tetrazolium]-1,3-benzene disulfonate) test (Roche, Mannheim, Germany). The assay was based on the cleavage of the water-soluble tetrazolium salt, WST-1, to water-soluble formazan as already reported [20]. For the test, cells were seeded in 96-well plates at a concentration of 10^4^ cells/100 µL CM and incubated with PFOA as above indicated. During the last 4 h of incubation, 20 µL of WST-1 reagent was added to cells, and the absorbance of the developed color was determined using the Victor Nivo at a wavelength of 450 nm with a reference length of 620 nm. The coefficients of variation were less than 3%.

##### Granulosa Cell Hydrogen Peroxide (H_2_O_2_) Production

For the test, cells were seeded in 96-well plates at a density of 2 × 10^5^ viable cells/200 μL CM and treated with PFOA as mentioned above. After centrifugation for 10 min at 400× *g*, the supernatant was discarded, and cells were lysed by adding cold Triton 0.5% + PMSF in PBS (200 μL/well) and incubating on ice for 30 min. H_2_O_2_ production was measured through a sensitive one-step analysis using the Amplex Red reagent; the kit used is the Amplex Red Hydrogen Peroxide Assay Kit (Molecular Probes, PoortGebouw, The Netherlands); the reagent reacts with H_2_O_2_ in the sample to produce the fluorescent oxidation product resorufin. Briefly, 5 µL of cell lysates were dispensed in each well of a 96-well plate and mixed with 45 µL of reaction buffer (0.05 M sodium phosphate, pH 7.4). Thereafter, 50 µL of Amplex Red reagent (100 µM)/HRP (0.2 U/mL) working solution were added to each microplate well, incubated at room temperature for 30 min, protected from light, and read against a standard curve of H_2_O_2_ ranging from 0.39 to 50 µM. Absorbance was determined with Victor Nivo using a 540 nm filter [22].

##### Granulosa Cell Nitric Oxide (NO) Production

In total, 2 × 10^5^ viable cells/200 μL CM were seeded in 96-well plates and treated with PFOA as previously detailed. Plates were then subjected to centrifugation for 10 min at 400× *g*; the test allowed for evaluating NO levels by measuring the nitrites in the supernatant of the samples using a microplate method based on the formation of an azo dye after the reaction with the Greiss reagent, which was prepared fresh daily by mixing equal volumes of 1% sulfanilamide, 5% phosphoric acid (stock solution 1) and 0.1% *N*-[naphtyl] ethylenediaminedihydrochloride (stock solution 2). After incubation with a Greiss reagent at room temperature, the colorimetric reaction was determined with the Victor Reader using a 540 nm against 620 nm filter. Furthermore, a standard curve between 25 and 0.39 μM was set up by diluting the 10 mM sodium nitrite in CM and on which the absorbances of the samples were interpolated [23].

##### Non-Enzymatic Scavenging Activity

The ferric reducing ability of plasma (FRAP) test is a direct measure of the total reducing power of a solution; is a colorimetric method that is based on the reduction, by antioxidant chemical agents present in the sample, in ferric-tripiridyltriazine (Fe^3+^ TPTZ) into a ferrous form (Fe^2+^ TPTZ). Fe^2+^ is measured spectrophotometrically by determining its colored complex with 2,4,6-tris(2-pyridyl)-s-triazine (Fe^2+^ TPTZ). The TPTZ reagent was prepared before use by mixing 25 mL of acetate buffer, 2.5 mL of 2,4,6-Tris(2-pyridyl)-s-triazine (TPTZ) 10 mM in HCl 40 mM, and FeCl_3_−6H_2_O solution. For the test, cells were seeded in 96-well plates at a density of 2 × 10^5^ cells/200 μL CM and treated with PFOA. Lastly, plates were subjected to centrifugation for 10 min at 400× *g*; supernatants were discarded, and cells were lysed by adding in an ice bath for 30 min cold Triton 0.5% + PMSF in PBS (200 μL/well). For the test, 40 μL of cell lysates added to Fe^3+^ TPTZ reagent are loaded in the wells. After 30 min incubation at 37 °C, absorbance of Fe^2+^ TPTZ was determined by Victor Reader at 595 nm. To quantify the absorbance, a standard curve must be prepared starting from a stock solution (1 mM) consisting of 0.0278 g of FeSO_4_ in 100 mL of distilled H_2_O, from which the other dilutions are prepared: 750 μM-500 μM-250 μM-100 μM [24]. 

### 2.2. Statistical Analysis

The experiments were repeated five times on the granulosa cells, each time starting from ovaries of about 40 gilts. Each experimental treatment was set up each time using mixed cells collected from all 40 gilts. Therefore, no animal effect was present in our experimental model. Each time, six replicates were made in cell cultures for controls and PFOA treatments. Data are presented as mean ± SEM. Statistical differences were calculated by One Way ANOVA considering treatment as the main factor using Statgraphics software (STC Inc., Rockville, MD, USA); in the presence of a significant difference (*p* < 0.05), the means were subjected to the Scheffè F test for comparisons multiples.

## 3. Results

### 3.1. Effect of PFOA on Swine Granulosa Cells Viability

**Figure 1:**
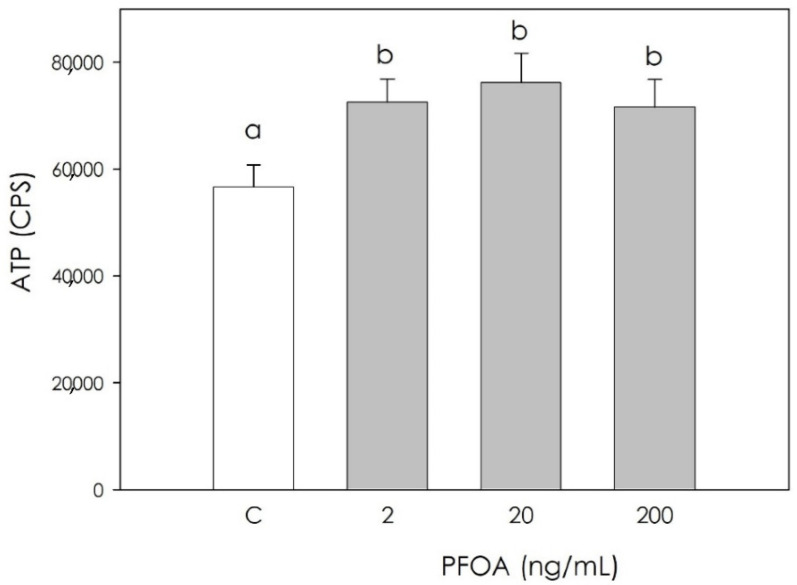
Effect of 48 h treatment with or without (C) perfluorooctanoic acid (PFOA) (2, 20 and 200 ng/mL) on cell metabolic activity quantified using ATP production in swine granulosa cell culture media. Data expressed as counts per second (CPS) represent the mean ± SEM of six replicates/treatment repeated in five different experiments. Different letters indicate a significant difference (*p* < 0.05).

All the examined PFOA concentrations significantly increased (*p* < 0.05) cell metabolic activity, evaluated as ATP production (Figure 1).

### 3.2. Effect of PFOA on Swine Granulosa Cells Redox Status

**Figure 2:**
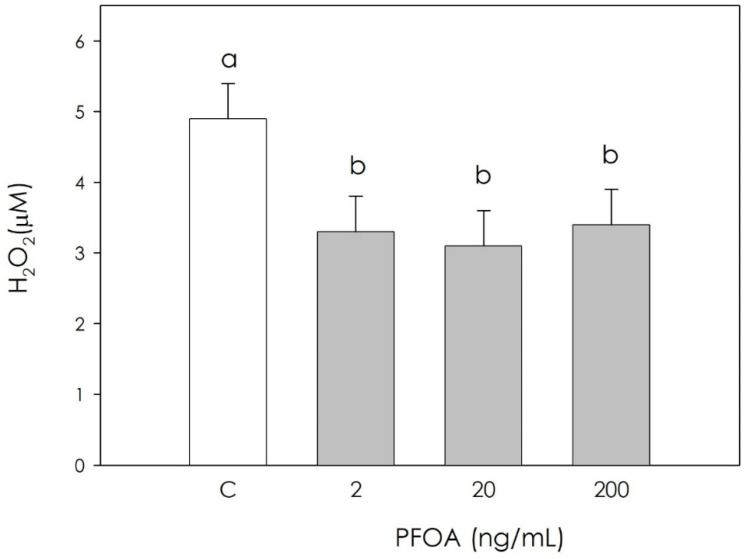
Effect of 48 h treatment with or without (C) perfluorooctanoic acid (PFOA) (2, 20 and 200 ng/mL) on hydrogen peroxide (H_2_O_2_) production in swine granulosa cell lysates. Data expressed as µM represent the mean ± SEM of six replicates/treatment repeated in five different experiments. Different letters indicate a significant difference (*p* < 0.05).

**Figure 3:**
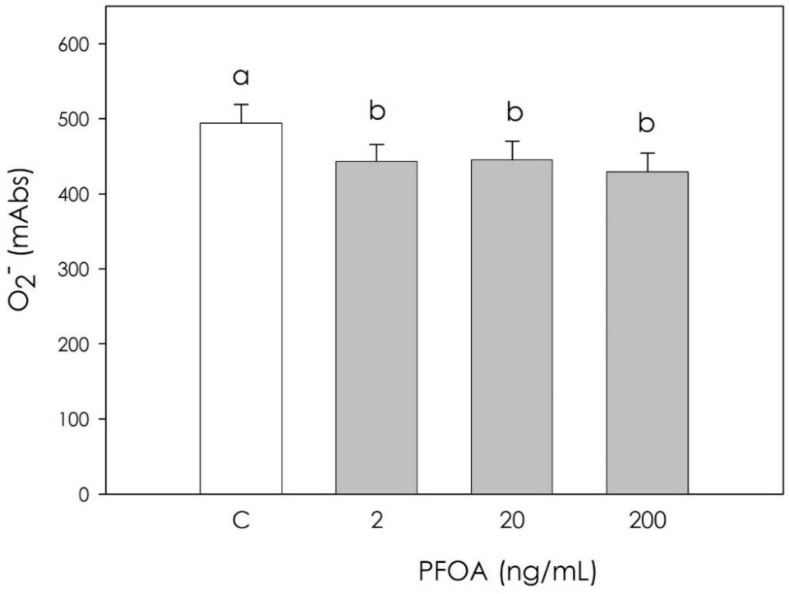
Effect of 48 h treatment with or without (C) perfluorooctanoic acid (PFOA) (2, 20, and 200 ng/mL) on superoxide anion (O_2_^−^) generation in swine granulosa cell culture media. Data expressed as milliabsorbance units (milliAbs), represent the mean ± SEM of six replicates/treatment repeated in five different experiments. Different letters indicate a significant difference (*p* < 0.05).

**Figure 4:**
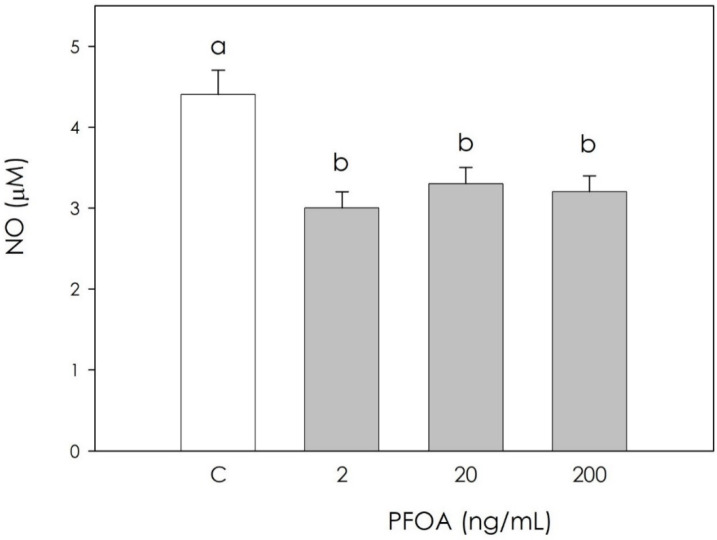
Effect of 48 h treatment with or without (C) perfluorooctanoic acid (PFOA) (2, 20 and 200 ng/mL) on NO production quantified using Griess reagent in swine granulosa cell culture media. Data expressed as µM represent the mean ± SEM of six replicates/treatment repeated in five different experiments. Different letters indicate a significant difference (*p* < 0.05).

In the production of free radicals, H_2_O_2_, O_2_^−^, and NO were significantly inhibited (*p* < 0.05) by PFOA at all examined concentrations (Figure 2, Figure 3 and Figure 4).

**Figure 5:**
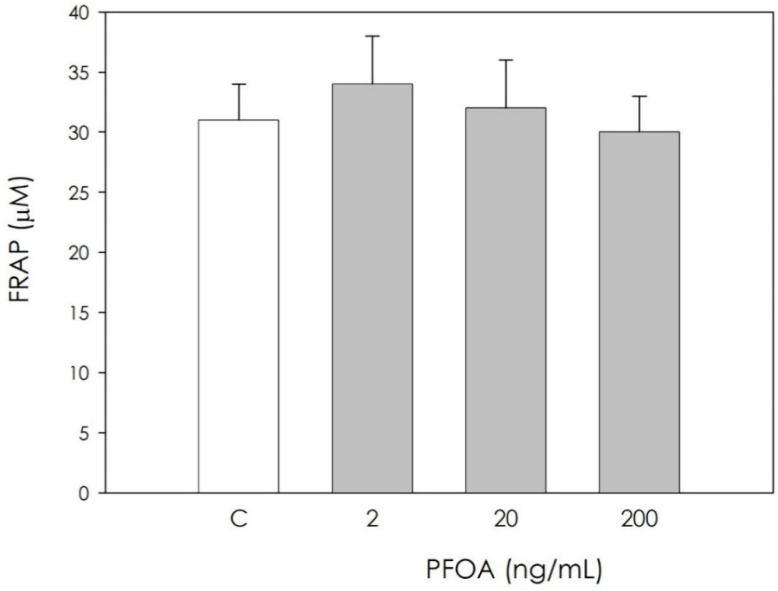
Effect of 48 h treatment with or without (C) perfluorooctanoic acid (PFOA) (2, 20 and 200 ng/mL) on nonenzymatic scavenger activity quantified using FRAP method in swine granulosa cell culture media. Data, expressed as µM, represent the mean ± SEM of six replicates/treatment repeated in five different experiments.

Scavenging activity represented by nonenzymatic antioxidant power was unaffected by PFOA (Figure 5).

## 4. Discussion

Perfluoroalkyl substances (PFAS) represents a broad group of artificial chemicals whose potential impact on human and animal health is gaining increasing attention and concern. Their peculiar chemical and physical properties have led to a massive use in many consumer and industrial products, with a resulting accumulation in both the environment and in organisms, mainly due to their strong resistance to degradation [4]. Particular concerns have been raised against perfluorooctanoic acid (PFOA), a “forever pollutant”. Due to long persistence, reproduction could be one of the main targets of its disruptive action [25]. Reproduction in mammals is in fact dependent on the hypothalamic–pituitary–gonadal axis, and can be modified by endogenous and exogenous signals. Within the ovary, coordinated interactions among theca cells, granulosa cells, and the oocyte are essential in follicular development, oocyte maturation, and ovulation. On this basis, this research was undertaken to explore the potential effect of PFOA on swine granulosa cells, a model of endocrine reproductive cells [11,12,19]. First, we investigated the PFOA effect on cell metabolic activity, evaluated as ATP production, and we found a significant increase. Krawczyk et al. [26] reported the effects of a mixture of endocrine disrupting chemicals (EDCs) including PFOA in human granulosa cells, documenting both increased mitochondrial activity and ATP content, which in turn is an index of cell viability. On the other hand, research on different cell types showed the inhibition of metabolic activity after exposure to PFOA: in mouse liver cells, the main PFOA target, Sun et al. [27] highlighted a decrease in cell viability, as assessed by ATP production after treatment with PFOA 200 μM for 28 days. Souders et al. [28] studied human neuron cells, observing that 24 and 48 h exposure to PFOA (400 µM PFOA) inhibits ATP production. In addition, exposure to PFOA 250 µM resulted in a decrease in ATP synthase activity. Since granulosa cell redox status is directly involved in the regulation of ovarian physiology follicular function regulation [14], we measured several oxidative stress markers in order to verify a potential impairment of follicular function by PFOA. Our results indicate that PFOA treatment significantly inhibited the production of superoxide anion, NO and hydrogen peroxide, while nonenzymatic antioxidant power was unaffected. Studies conducted on the modulation of oxidative stress induced by PFOA in the ovary are limited. Divergence between the few available results in the literature and our current observations may depend on differences in the experimental model, and various times of exposure to the substance and different concentrations of PFOA. Specific studies on the interaction of PFOA with ovarian cells are limited. Among these, a study conducted in 2019 by Lopez-Arellano et al. [29] on mouse oocytes exposed to PFOA (50, 100, and 150 μM) for 24 h documented that ROS levels increased significantly and concentration-dependently. These results agree with those obtained in an in vivo study [30] that reported that PFOA treatment inhibited the activities of superoxide dismutase and catalase, and increased the generation of hydrogen peroxide and malondialdehyde in the ovaries of pregnant mice. A recent study on cumulus oophore cells (oocyte and granulosa cells) documented that exposure to 20 and 40 μM PFOA for 44 h significantly stimulated ROS production [31]; the observed increase in ROS was attributed to antioxidant system damage, as already reported in a study by Xu et al. [32], possibly leading to the lipid peroxidation of mitochondrial membranes. This would result in ion losses, which can affect the proton gradient and oxidative phosphorylation required to produce ATP, thus contributing to the further generation of ROS [33].

## 5. Conclusions

This research demonstrated that PFOA disrupts the metabolic activity and redox status of swine ovarian cells. In conclusion, collected data from the present research added to the limited knowledge on PFOA interaction with the reproductive system, and may provide a stimulus to continue the evaluation of the potentially critical effects of this substance. Further studies are needed to better explore the effect of PFOA in granulosa cells, in particular to verify its potential effects on steroidogenic activity.

## Data Availability

Data are available upon request.
